# Flexible Coding of Morpheme Position Information in Tibetan Reading: Evidence from Eye Movements

**DOI:** 10.3390/bs16091635

**Published:** 2026-09-13

**Authors:** Shan Li, Chunhui Zhang, Lei Gao, Chengzhi Wang, Xiaolei Gao

**Affiliations:** 1 Plateau Brain Science Research Center, Xizang University, Lhasa 850000, China; lishan202297@163.com (S.L.); zchunhui2001@163.com (C.Z.); gaolei1983good@163.com (L.G.); 2 School of Psychology, Liaoning Normal University, Dalian 116029, China; 3 Key Laboratory of Brain and Cognitive Neuroscience, Institute of Psychological and Brain Sciences, Liaoning Normal University, Dalian 116029, China; wcz9803@163.com

**Keywords:** Tibetan reading, morpheme position information, transposed-letter effect, lexical access, eye movement

## Abstract

Morpheme position information, referring to the relative sequential order of morphemes within a word, is crucial for word recognition and reading comprehension. Tibetan combines alphabetic and ideographic features, but it remains unclear whether morpheme position information is processed in Tibetan reading, whether the transposed-letter effect manifests itself, and how lexical access is represented for Tibetan two-character words. We conducted two eye-tracking experiments to address these questions. In Experiment 1, in the identical condition, all temporal eye-movement measures were the shortest, with the largest progressive saccade amplitude and the fewest number of fixations. The transposed condition ranked second, whereas the substituted condition resulted in the longest temporal measures, the smallest progressive saccade amplitude, and the greatest number of fixations. Experiment 2 showed a consistent pattern with Experiment 1, and no interaction was observed between presentation type and word frequency. The two experiments jointly indicated that morpheme position information is processed during Tibetan reading. Transposed nonwords activate the base word more readily than substituted nonwords, suggesting that morpheme decomposition serves as the key pathway for word recognition. This study provides the first empirical evidence for morpheme position information processing and the transposed-letter effect in Tibetan reading, supporting the decomposed morphological representation view.

## 1. Introduction

Reading is a vital and complex cognitive activity, requiring readers to integrate information from characters and words to achieve comprehension at the sentence and discourse levels. As the primary stage of reading, word recognition is a cognitive psychological process involving the decoding and encoding of information contained within words to construct meaning (Sandra, 1994). Within this process, the morpheme is the smallest phonetic–semantic unit and linguistic unit that can be used independently and can distinguish meaning (C. Wang & Peng, 2000). Polymorphemic words, formed by combining two or more morphemes, are a dominant and common pattern of word formation in language (Dressler, 2010; Ding & Peng, 2006; Y. Wu & Li, 2018; J. Wang et al., 2014). The recognition of polymorphemic words mainly involves two key processes: the processing of morpheme identity information (identifying the specific morphemic components that constitute the word), and the processing of morpheme position information (identifying the relative order of different morphemes within the word) (Cao et al., 2023; Gu & Shi, 2021; Hua et al., 2017). Research has confirmed that the processing of morpheme position information is an indispensable component of both word recognition and reading comprehension (Gu et al., 2015; Hua et al., 2017; Peng et al., 1999). If readers fail to effectively process morpheme position information, they cannot accurately distinguish between words (e.g., “蜜蜂” and “蜂蜜” in Chinese, “bookcase” and “casebook” in English), consequently impairing sentence and discourse comprehension.

Previous studies on the processing of morpheme position information have found the transposed-letter effect (TL effect) (Baciero et al., 2022; Gómez et al., 2025; Johnson et al., 2007; Perea et al., 2023). This effect refers to the phenomenon where transposed letters (TL nonwords, where two letters in a word are transposed, e.g., “jugde”) better facilitate activation and recognition of the base word (e.g., “judge”) than substituted letters (SL nonwords, where two letters in a word are substituted with other letters, e.g., “jupbe”) (Christianson et al., 2005; Johnson & Dunne, 2012; Perea & Lupker, 2003; Perea et al., 2016). The TL effect indicates that character position information is encoded to some extent, but not in an absolutely strict manner. In other words, there is flexibility in character position encoding and it is not completely fixed (Blythe et al., 2014; Duñabeitia et al., 2014; Velan et al., 2013; Winskel et al., 2012). Consequently, although transposed characters do hinder reading comprehension to some extent, their disruptive impact is significantly less severe than that of substituted characters (Gu et al., 2022; Perea & Fraga, 2006; Perea et al., 2012). For both types of nonwords, the positions of two characters are incorrect. If character position encoding is strictly enforced, the activation levels of TL nonwords and SL nonwords for the base word should be no different.

The TL effect has been widely validated. In studies of alphabetic systems (e.g., English, Spanish), Rayner et al. (2006) asked participants to read sentences containing transposed initial letters, transposed internal letters, transposed final letters, and no transpositions. Results showed that all conditions containing transposed letters resulted in decreased reading efficiency. In Chinese studies, for instance, Bian et al. (2010) found that when sentences contained transposed two-character words, the reading time of participants significantly increased, and all eye-tracking measures were affected. This indicated that the transposition of morpheme position information affects reading.

However, notably, some researchers have pointed out that the TL effect may have language specificity, whether it exists stably remains undetermined. Research has found that transposed characters in Hebrew significantly impaired the performance of Hebrew-English bilingual participants, whereas transposed characters in English had almost no effect on the participants (Velan & Frost, 2007). Researchers suggested that the different reading performances produced by transposed character in these two languages are caused by the differences in lexical organization between the languages, which may potentially be related to the root-affix structure and rich morphological changes in Hebrew. In addition, Rastle et al. (2019) used Korean as the experimental material, and observed no priming effect caused by identical characters or syllables in different positions, indicating that there is no flexibility in processing character position information during Korean reading. They proposed that although Korean is a phonetic script, it essentially features a high degree of orthographic ambiguity. Its written form incorporates the square character structure of Chinese characters. It is possibly due to the differences in script properties between Korean and English or Chinese that affect the precision of orthographic representation. Consequently, the TL effect was not observed in Korean reading.

In summary, although a robust TL effect has been found in representative alphabetic writing systems (e.g., English, Spanish) as well as in Chinese (the ideographic writing system), it has not been observed in some special languages, such as Hebrew and Korean. This suggests that the TL effect may be modulated by linguistic features (such as orthographic structure, writing direction, and orthographic depth), indicating a degree of language specificity. Whether the TL effect stably exists across different writing systems remains unclear and requires further empirical investigation.

Moreover, another important issue is the representational format of polymorphemic words. Specifically, how does morpheme position information play a role in and affect word recognition? There are three controversial viewpoints on this: the morpheme access view, the whole-word access view, and the dual-route access view (Aini et al., 2023; Cao et al., 2023; C. Wang & Peng, 2000; Yu, 2017). The morpheme access view posits that polymorphemic words are represented in a decomposed manner in the mental lexicon, such that morpheme recognition is a necessary way for whole-word access and entails the processing of morpheme information (including identity and position) (Bergman et al., 1988; Taft & Forster, 1975; Zhao, 2015). In contrast, the whole-word access view proposes that the mental lexicon stores only whole-word representations, such that word recognition involves directly accessing the corresponding whole-word entry. This view therefore denies the existence of separate morpheme representations or an independent stage for processing morpheme position information (Butterworth, 2011; Cole et al., 1989; Lukatela et al., 1980). According to the dual-route access view, during the lexical access of polymorphemic words, both morphemes and whole-word units play a role, with neither being the exclusive pathway. Consequently, lexical processing is viewed as the outcome of their combined action. Meanwhile, the access route for polymorphemic words is modulated by factors such as word frequency. Specifically, high-frequency words may be accessed as whole units, whereas low-frequency or novel words tend to be processed via morphemic access (Kuperman et al., 2009; Marslen-Wilson et al., 1994; Taft, 1994; Xiao & Yang, 2014).

To address the aforementioned controversies, studies have been conducted on different languages from various perspectives.

In studies on alphabetic writing systems (such as English, German, Finnish, Dutch, Basque, Spanish, etc.) (Bien et al., 2005; Hyönä & Pollatsek, 1998), Bronk et al. (2013) employed a lexical decision task and found that participants responded significantly faster to German polymorphemic words containing high-frequency morphemes than to those with low-frequency morphemes, supporting the morpheme access view. Conversely, other studies have shown that gaze duration is significantly influenced by whole-word frequency but not by morpheme frequency, consistent with the whole-word access view (Pollatsek et al., 2000). Furthermore, research combining behavioral and eye-tracking measures has demonstrated that both the frequency of initial and final morphemes, and whole-word frequency influence lexical processing. Specifically, long, novel, and low-frequency words appear to be accessed via their constituent morphemes, whereas short, familiar, and high-frequency words are accessed as whole units. This pattern of results indicates that polymorphemic words may be recognized through a dual-route access mechanism (Bertram & Hyönä, 2003; Duñabeitia et al., 2007).

Research on Chinese polymorphemic word processing has similarly yielded mixed findings. Some studies have found that participants recognize polymorphemic words more easily when both the initial and final morphemes are of high frequency, confirming the role of morpheme position information and supporting the morpheme access view (Forster & Taft, 1994; Bian et al., 2010). In contrast, some eye-tracking experiments have observed only the word-frequency effect with no character-frequency effect, which aligns with the whole-word access view (Ma et al., 2015). Furthermore, other studies manipulating the frequencies of initial/final characters and whole words have found that high-frequency words tend to be represented as whole units, whereas low-frequency words rely more on morpheme decomposition, and that the character-frequency effect is modulated by word frequency, providing evidence for the dual-route access view (Yan et al., 2006).

In summary, there is still no consensus on whether polymorphemic words are accessed via morphemic, whole-word, or dual-route representations. This lack of agreement persists across different writing systems (alphabetic or ideographic) and methodological approaches (whether applied within a single language or across languages). Given these persistent theoretical disagreements, the fundamental issue of how polymorphemic words are represented in lexical processing warrants further investigation.

Tibetan is a phonetic writing system belonging to the Tibeto-Burman branch of the Sino-Tibetan language family (Bai et al., 2017). It is a unique script that combines features of both alphabetic and ideographic systems. Compared with English and Chinese, Tibetan shares the following similarities and differences: (1) Similar to alphabetic systems, Tibetan is composed of letters and has a linear writing structure, yet its character structure exhibits a three-dimensionality like Chinese. (2) Similar to Chinese, Tibetan text is composed of sentences forming a continuous passage without explicit boundaries between words. Consequently, word recognition lacks effective and accurate marks, and sentence comprehension requires readers to segment words through their mental lexicon and then proceed based on the relationships between words. (3) In Chinese, there are no marks between characters or words, while in English, spaces are used between words. However, in Tibetan, the tsheg “་” (syllable divider) and shad “།” (sentence marker) are used as marks between characters and sentences (Li et al., 2022a; D. Wang et al., 2023). In Tibetan, morphemes are represented as “syllabic characters” composed of phonemes, known as “Tibetan characters” (F. Wu, 2022). Within the Tibetan lexical system, two-character polymorphemic words constitute the largest portion (approximately 55.29%) of the vocabulary, serving as a core component and unit (F. Wu, 2022; Zhu, 1983). These combined features make Tibetan a valuable test case for examining whether morpheme position processing is universal or language specific. Specifically, Tibetan differs from both alphabetic scripts and Chinese in ways that matter for morpheme position processing. Unlike English, Tibetan uses the tsheg “་” to mark syllable boundaries, providing visible cues that might reduce the need for morpheme-level segmentation (Li et al., 2022a; D. Wang et al., 2023). Yet unlike Chinese, where each character is a monosyllabic morpheme, Tibetan two-character words contain two morphemes separated by a tsheg (Shen et al., 2023; Liang et al., 2026). This raises a key question: if morpheme transposition disrupts reading even when the orthography already marks clear sub-lexical boundaries, it would suggest that morpheme position processing is mandatory and universal; if not, it would depend on orthographic transparency. Moreover, because Tibetan has no inter-word spaces (like Chinese), readers must simultaneously segment words and process morpheme structure, providing a unique opportunity to disentangle the contributions of orthographic and morphological structures—a distinction that remains conflated in most existing research. Most existing models of morpheme processing have been built on a limited set of well-studied languages (e.g., English, German, Finnish, and Chinese). Tibetan, as a Tibeto-Burman language, offers a crucial test of whether these models generalize beyond the language families on which they were developed. By examining transposed-morpheme effects in Tibetan, we can isolate the contribution of morphological structure from confounding orthographic factors. Thus, investigating morpheme position processing in Tibetan not only extends the empirical database to an underrepresented language family but also informs the broader theoretical debate on whether polymorphemic word recognition proceeds via whole-word storage, morpheme decomposition, or a dual-route mechanism.

In conclusion, many empirical studies have supported the role of morpheme position information processing in word recognition and its flexibility, with the TL effect being widely observed (Gu et al., 2015; Mizuno & Matsui, 2013; Perea et al., 2016; Winskel et al., 2012). However, other studies have shown that for certain writing systems with unique features, the processing of morpheme position information may be more rigid, with no TL effect observed, suggesting that this effect may exhibit a degree of language specificity (Rastle et al., 2019; Velan & Frost, 2007). Tibetan, as a language with distinctive morphological and word formation rules, remains largely unexplored. This begs the question whether morpheme position information is processed in Tibetan reading, and whether the TL effect can be observed. Given the unique orthography and morphology of Tibetan, it is unclear whether its processing pattern of morpheme position information shares commonalities with both alphabetic and Chinese writing systems, or exhibits a distinctive pattern of its own. Furthermore, the existing lexical-access accounts make different predictions regarding the recognition of Tibetan two-character words, particularly as a function of word frequency. These issues remain unresolved. To address these gaps and theoretical debates, the present study aimed to investigate the following research questions: (1) Do Tibetan readers process morpheme position information during word recognition, as evidenced by a TL effect? (2) Does the processing pattern of morpheme position information in Tibetan share commonalities with alphabetic and Chinese writing systems, or does it exhibit a unique pattern due to its distinctive orthographic and morphological features? (3) Which theoretical perspective on lexical access is supported by the processing of Tibetan two-character words, and does morpheme position information affect the recognition of two-character words with different word frequencies?

Therefore, the present study conducted two eye-tracking experiments, selecting the most representative two-character words in the Tibetan lexicon. By transposing the two syllabic characters separated by a tsheg “་”, we aimed to investigate the effect of morpheme position information on lexical processing during Tibetan reading. In Experiment 1, we manipulated the presentation of two-character words within sentences and recorded participants’ eye movements while they read sentences containing morpheme transpositions. Experiment 1 examined whether morpheme transposition affects Tibetan reading and whether the TL effect is observed during Tibetan reading. Since polymorphemic words consist of multiple morphemes, their access processes may be jointly influenced by whole-word frequency and morpheme frequency at the same time. Thus, investigating the representational format of polymorphemic words access in Tibetan reading can be directly approached by manipulating both morpheme position information and word frequency. Based on Experiment 1, Experiment 2 introduced word frequency as an additional variable to further examine whether morpheme transposition affects the processing of morpheme position information in Tibetan two-character words with different word frequencies, thereby providing empirical evidence on the representational format of Tibetan two-character word access. This study hypothesized that in Tibetan reading, morpheme transposition would affect processing, demonstrate reliance on morpheme position information, and manifest the TL effect. The results were expected to support the morpheme access view of polymorphemic word recognition.

## 2. Experiment 1

In Experiment 1, we manipulated the presentation of target words to investigate the effect of morpheme transposition on Tibetan sentence reading. This experiment aimed to explore whether Tibetan reading involves the processing of morpheme position information and whether the TL effect exists.

### 2.1. Materials and Methods

#### 2.1.1. Participants

To ensure sufficient statistical power, with reference to previous studies (Huang et al., 2021; Faul et al., 2007), the sample size was estimated using G*Power3.1.9.2 before the experiment (*F* tests, ANOVA: repeated measures, within factors; α = 0.05, 1 − β = 0.95, correlation among repeated measures *r* = 0.5, number of groups = 1, number of measurements = 3, nonsphericity correction ε = 1). Based on a medium effect size (*f* = 0.25), the analysis suggested that 45 participants would be required. To account for potential data loss, 60 Tibetan college students (mean age = 20.37 ± 1.56 years; 41 females, 19 males) were recruited. All participants met the following inclusion criteria: (a) native Tibetan speakers; (b) normal or corrected-to-normal vision; (c) no diagnosis of dyslexia or other reading-related disorders. Regarding linguistic background, all participants reported acquiring Tibetan from birth (age of acquisition ≥ 3 years), receiving at least 10 years of formal Tibetan-language education, and beginning Chinese literacy instruction in approximately Grade 3. Tibetan was the primary language used in daily communication, at home, and throughout formal education (primary school through university). Self-reported proficiency ratings on a 5-point scale (1 = not at all proficient, 5 = very proficient) showed that Tibetan proficiency was significantly higher than Chinese proficiency: overall level (Tibetan: *M* = 4.22, *SD* = 0.61; Chinese: *M* = 3.80, *SD* = 0.66), speaking (Tibetan: *M* = 4.34, *SD* = 0.68; Chinese: *M* = 3.73, *SD* = 0.73), listening (Tibetan: *M* = 4.36, *SD* = 0.60; Chinese: *M* = 3.92, *SD* = 0.61), reading (Tibetan: *M* = 4.31, *SD* = 0.62; Chinese: *M* = 3.86, *SD* = 0.75), and writing (Tibetan: *M* = 4.20, *SD* = 0.63; Chinese: *M* = 3.75, *SD* = 0.75). Participants also reported using Tibetan more frequently than Chinese in daily life (daily use percentage: Tibetan *M* = 71.36%; Chinese *M* = 28.64%). These data confirm that Tibetan was the dominant language for all participants. All participants were unaware of the specific experimental hypotheses (they were informed that the study concerned Tibetan reading comprehension). All provided written informed consent prior to the experiment and received monetary compensation upon completion. This study was approved by the Ethics Committee of Xizang University (IRB NO: XZDXLL2022016).

#### 2.1.2. Design

A single-factor with three-level (presentation type: identical, transposed, substituted) within-participant design was employed.

#### 2.1.3. Experimental Materials

(1) Selection and compilation of materials

Firstly, referring to the previous literature (Li et al., 2022a), we selected sentences from upper elementary school Tibetan textbooks and comparable level extracurricular books. Seventy-eight Tibetan two-character words were used as target words. Secondly, a corresponding sentence frame was constructed for each target word, positioning it near the middle of the sentence. Each target word was embedded in its frame and presented under three presentation types: ① identical (the target word itself); ② transposed (a non-word created by transposing the positions of its two characters); ③ substituted (a non-word formed by replacing two characters of the target word with other unrelated characters).

To prevent transposition or substitution of characters from interfering with word segmentation and thus sentence comprehension, it was specified when compiling the sentence that the characters of the target word could not form a real word with adjacent characters in the sentence, nor could the transposed characters form a real word. Under transposed and substituted conditions, each morpheme was not allowed to form a real word with adjacent characters.

All target words were nouns. Their horizontal word length (i.e., linear phoneme count) ranged from 2 to 7 phonemes, and their total word length (i.e., spatial phoneme counts in the three-dimensional Tibetan script) ranged from 2 to 9 phonemes. The transposed and substituted non-words were matched to their base words in horizontal length, total word length and (for substituted items) character morphology. All sentences were single-line declarative sentences, with the horizontal sentence length ranging from 14 to 39 phonemes, and the total sentence length ranging from 23 to 61 phonemes. The sentences contained no semantic or syntactic ambiguities and no punctuation. An example of the materials is presented in Table 1.

(2) Evaluation and arrangement of materials

First, 15 Tibetan university students who did not participate in the formal experiment were invited to rate the familiarity of 78 target words on a 5-point scale (1 = very familiar, 5 = very unfamiliar). Another 15 Tibetan university students who did not participate in the formal experiment rated the difficulty and naturalness of 78 sentence frames on a separate 5-point scale (difficulty: 1 = very easy, 5 = very difficult; naturalness: 1 = very natural, 5 = very unnatural). Based on these ratings, 6 sentences were assigned to a practice block and 72 to the formal experimental block. The selected experimental materials showed high familiarity, easy, and high naturalness (familiarity: *M* = 1.40, *SD* = 1.62; difficulty: *M* = 1.66, *SD* = 0.74; naturalness: *M* = 1.40, *SD* = 0.70).

To control for predictability, a separate group of 15 Tibetan university students who did not take part in the formal experiment were invited to complete a cloze test. They were presented with the sentence frames with the target word omitted and were asked to provide the first word that came to mind. A word was considered as high predictable if >75% of the participants supplied it, and as low predictable if <20% did. The average cloze predictability was 2.96% (*SE* = 0.01), confirming that the target words were low predictable.

Thus, the final materials met the requirements of high familiarity, low difficulty, high naturalness, and low predictability. The three presentation types of target words (identical, transposed, substituted) were embedded into the 72 sentence frames, ensuring that all parts of sentences except for the target words were identical. This process ultimately yielded 216 (3 × 72) Tibetan sentences (horizontal word length: *M* = 4.43; total word length: *M* = 6.69; horizontal sentence length: *M* = 25.65; total sentence length: *M* = 38.11).

To prevent order and repetition effects, the three experimental conditions were distributed across three lists using a Latin square design. Each participant was assigned to read only one list and did not encounter the same sentence frame more than once. Each list contained 72 trials (24 trials per condition), presented in a randomized order.

#### 2.1.4. Apparatus

Eye movements were recorded using an SR Research EyeLink1000 Plus eye-tracking system (SR Research Ltd, Ottawa, ON, Canda) with a sampling rate of 1000 Hz. The stimuli were presented on a 24.5-inch Dell monitor (screen resolution: 1920 × 1080 pixels; refresh rate: 240 Hz; Dell Technologies, Round Rock, TX, USA). The sentences were displayed as a single line of black text in 36-point Microsoft Himalaya font against a white background. The participants were seated approximately 65 cm from the monitor. Each Tibetan character corresponded to approximately 0.6 degrees of visual angle at this viewing distance.

#### 2.1.5. Procedure

Participants were tested individually. Upon arriving at the laboratory, they were first familiarized with the environment and seated in a designated position. Then, they were given experimental instructions and a brief description of the apparatus, and were instructed to minimize head movements as much as possible throughout the session to ensure data quality. Subsequently, a three-point calibration and validation procedure was performed. Calibration was repeated until the average error fell below 0.25° of visual angle (Li et al., 2022b). Recalibration was conducted as needed (e.g., after a break or if drift exceeded 0.25°).

Following successful calibration, on-screen instructions were presented. After the participants read them, we explained the task requirements in more detail. The participants then completed a practice block of 6 sentences to ensure familiarity with the procedure. Each trial in the formal experiment began with a fixation point aligned to the position of the first character on the left side of the screen. The participants were required to fixate on this point and press a button to proceed. The sentences were then presented one at a time on a single line. The participants read at their natural pace.

**Figure 1:**
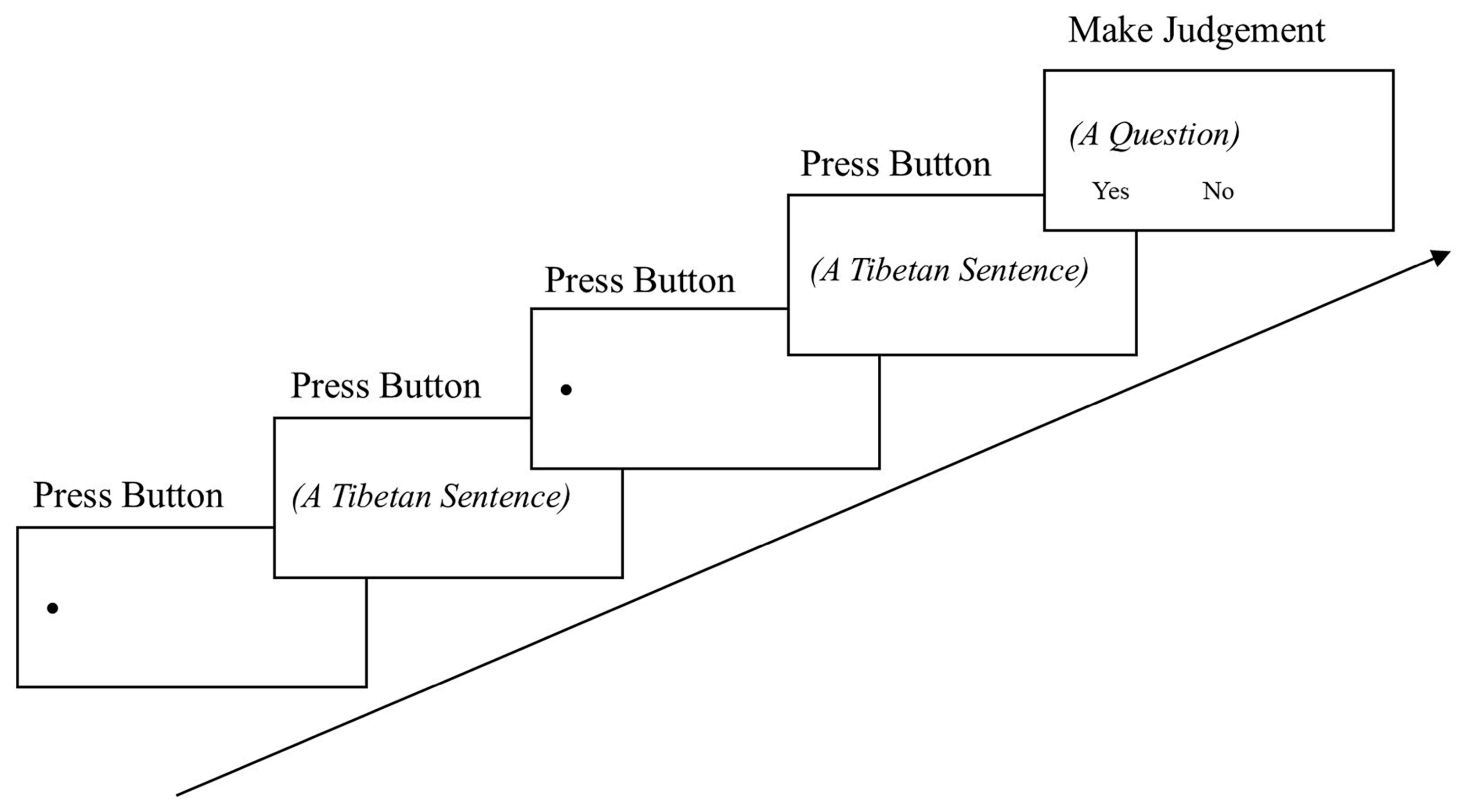
Procedure of Experiment 1.

To encourage attentive reading, 22 comprehension questions (with a balanced yes/no judgment) followed a subset of sentences, to which participants responded via key press. If a break was needed, the experiment was paused and recalibration was performed before resuming. The entire experiment lasted approximately 20 min. The experimental procedure is presented in Figure 1.

#### 2.1.6. Measures

With reference to the previous literature (Cao et al., 2023; Chang et al., 2020), the following global eye-movement measures (reflecting overall sentence processing) were analyzed: (a) regression path duration (RPD, the summed duration of all fixations on a sentence from the first fixation until the point of fixation moves to the right of the current area of interest, excluding the fixation point on this right area); (b) mean fixation duration (MFD, the average duration of all fixations on a sentence); and (c) progressive saccade amplitude (PSA, the length between two consecutive fixations). RPD not only reflects the process of lexical access but also indicates the later stages of lexical processing and semantic integration; MFD reflects the overall processing during reading; and PSA indicates the reader’s perceptual span during comprehension.

With reference to the previous literature (Cao et al., 2023; Chang et al., 2020; Gu et al., 2020), the following local eye-movement measures were analyzed for the two-syllable target word, which serves as the area of interest (AOI) in Experiment 1: (d) first fixation duration (FFD, the duration of the first fixation on the AOI during first-pass reading); (e) gaze duration (GD, the sum of all fixations on the AOI from first entering the region until leaving it during first-pass reading); (f) total fixation duration (TFD, the sum of all fixations on the AOI); and (g) fixation count/number of fixations (FC, the total number of fixations on the AOI). FFD and GD are sensitive to the early stage of lexical access; TFD reflects the overall process of word processing; and FC is an indicator of late-stage processing efficiency.

### 2.2. Results

The mean comprehension accuracy across all participants was 85.53%, indicating that they read the sentences attentively and with good comprehension, thus meeting the requirements of the experiment. Based on the previous literature (Cao et al., 2023; Li et al., 2022a), the criteria for the exclusion of data were the following: (1) trials with track loss or major tracking errors (due to the participants’ head movements and other factors in the experiment); (2) fixations shorter than 80 ms or longer than 1200 ms; and (3) trials in which the outlier exceeded three standard deviations. In total, 7.01% of all trials were removed.

Linear mixed models (LMM) were conducted using the lme4 package (Version 1.1-34; Bates et al., 2015) in R (Version 4.3.1; R Core Team, 2023) to analyze the data. Presentation type was included as a fixed factor. Participants and items were treated as crossed random effects, with by-participant and by-item random intercepts and slopes. For each eye-movement measure, we began with a model containing the maximal random-effects structure. If this model failed to converge, the model was progressively trimmed until convergence was achieved (Barr et al., 2013). All measures were log-transformed to better meet the assumption of normality. The pattern of results was virtually identical for untransformed and log-transformed data. Results are reported with regression coefficients (*b*), standard errors (*SE*), *t*-values, and 95% confidence intervals. Effects with |*t*| > 1.96 were considered statistically significant (*p* < 0.05). The final model structure for each eye-movement measure, along with the full model comparison results (including AIC values and likelihood ratio tests), is provided in Appendix A Table A1.

#### 2.2.1. Global Analysis

**Figure 2:**
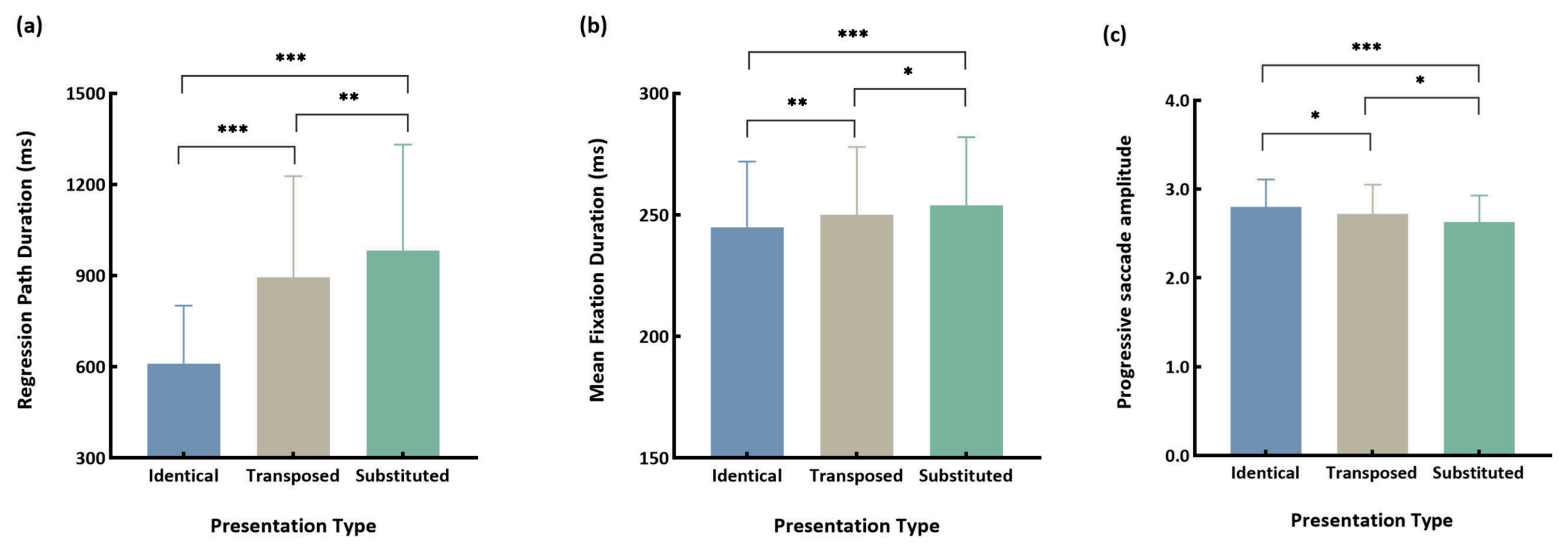
Statistical analysis results of eye-movement measures in global analysis of Experiment 1. (**a**) regression path duration, RPD; (**b**) mean fixation duration, MFD; (**c**) progressive saccade amplitude. PSA. *** *p* ≤ 0.001, ** *p* ≤ 0.01, * *p* ≤ 0.05. Error bars represent *SD*. The same applies below.

Descriptive statistics of eye-movement measures under different presentation types are shown in Table 2, with the corresponding statistical analysis results presented in Table 3 and Figure 2.

The global analyses revealed that, RPD and MFD in the substituted condition were significantly longer than those in both the identical condition (RPD: *t* = 12.73, *p* < 0.001; MFD: *t* = 5.32, *p* < 0.001) and the transposed condition (RPD: *t* = 3.16, *p* < 0.01; MFD: *t* = 2.35, *p* = 0.02); RPD and MFD in the transposed condition were significantly longer than those in the identical condition (RPD: *t* = 9.55, *p* < 0.001; MFD: *t* = 2.97, *p* < 0.01). PSA in the substituted condition was significantly shorter than that in both the identical condition (*t* = −4.13, *p* < 0.001) and the transposed condition (*t* = −2.13, *p* = 0.04); PSA in the transposed condition was significantly shorter than that in the identical condition (*t* = −2.02, *p* = 0.05).

#### 2.2.2. Local Analysis

**Figure 3:**
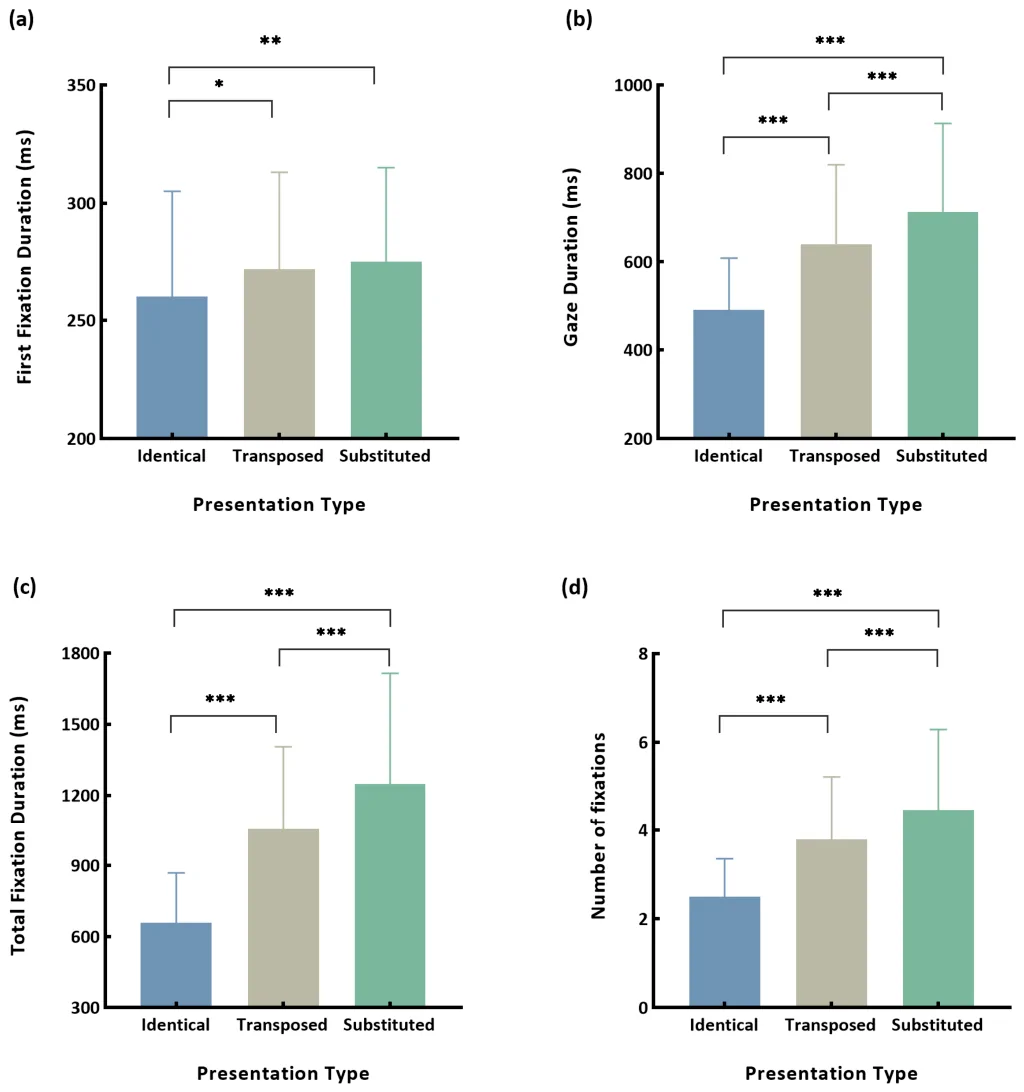
Statistical analysis results of eye-movement measures in local analysis of Experiment 1. (**a**) first fixation duration, FFD; (**b**) gaze duration, GD; (**c**) total fixation duration, TFD; (**d**) number of fixations, FC. *** *p* ≤ 0.001, ** *p* ≤ 0.01, * *p* ≤ 0.05.

Descriptive statistics of eye-movement measures under different presentation types are shown in Table 4, with the corresponding statistical analysis results presented in Table 5 and Figure 3.

The local analyses showed that FFDs in both the substituted condition (*t* = 2.69, *p* < 0.01) and the transposed condition (*t* = 2.03, *p* = 0.05) were significantly longer than FFD in the identical condition, and there was no significant difference between the substituted and transposed conditions for FFD (*t* = 0.65, *p* = 0.52). GD and TFD in the substituted condition were significantly longer than those in both the identical condition (GD: *t* = 11.20, *p* < 0.001; TFD: *t* = 16.65, *p* < 0.001) and the transposed condition (GD: *t* = 3.51, *p* < 0.001; TFD: *t* = 4.40, *p* < 0.001); GD and TFD in the transposed condition were significantly longer than those in the identical condition (GD: *t* = 7.67, *p* < 0.001; TFD: *t* = 12.23, *p* < 0.001). For FC, the substituted condition had more fixations than both the identical condition (*t* = 16.39, *p* < 0.001) and the transposed condition (*t* = 4.40, *p* < 0.001); the transposed condition had more fixations than the identical condition (*t* = 11.96, *p* < 0.001).

The combined global and local results of Experiment 1 consistently showed that in the identical conditions, temporal eye-movement measures (RPD, MFD, FFD, TFD) were the shortest, with the largest PSA, and the fewest fixations (FC), followed by the transposed condition. However, in the substituted condition, all temporal measures were the longest, with the smallest PSA and the highest number of fixations. This pattern indicated that the participants encoded morpheme position information during Tibetan sentence reading, and that incorrect morpheme position information interfered with processing, demonstrating that morpheme position information is actively processed in Tibetan reading. In addition, compared to the substituted condition, the transposed condition showed shorter RPD, MFD, FFD, and TFD, along with larger PSA and fewer fixations. This suggested that the participants had a certain flexibility in processing morpheme position information, and that transposed nonwords were more likely to activate the base word than substituted nonwords. This further suggested the presence of a TL effect in Tibetan reading. Therefore, the results of Experiment 1 did not support the whole-word access view, and indicated that morpheme position information was involved in the processing of Tibetan words. Building on these findings, Experiment 2 introduced the variable of word frequency to further examine the effect of morpheme transposition on the processing of Tibetan two-character words with different frequencies, thereby aiming to reveal the lexical access representation of Tibetan two-character words.

## 3. Experiment 2

In Experiment 2, we examined how transposed morphemes affect the processing of Tibetan two-character words by manipulating both word frequency and presentation type, in order to investigate the representational model underlying lexical access for Tibetan two-character words.

### 3.1. Materials and Methods

#### 3.1.1. Participants

As in Experiment 1, the sample size was estimated using G*Power3.1.9.2 before the experiment (*F* tests, ANOVA: repeated measures, within factors; α = 0.05, 1 − β = 0.95, correlation among repeated measures *r* = 0.5, number of groups = 1, number of measurements = 4, nonsphericity correction ε = 1). This setup, treating the four cells of the 2 × 2 design as four repeated measurements, was used to approximate the power for the word frequency × presentation type interaction. G*Power does not currently provide a dedicated procedure for a priori sample size estimation for LMMs with many repeated time points (Ascic et al., 2026). We therefore adopted the commonly used strategy of approximating the design with a repeated-measures ANOVA (G*Power, for an a priori power analysis) during the experimental planning stage. The two statistical models are not identical, but considering that LMMs generally have higher statistical power than repeated-measures ANOVA, the G*Power calculation based on the ANOVA framework provides a conservative proxy for our LMM-based analysis. Based on a medium effect size (*f* = 0.25), the analysis suggested that 36 participants would be required. To account for potential data loss, 64 Tibetan college students (mean age = 20.34 ± 1.49 years; 40 females, 24 males) were recruited. The inclusion criteria and self-reported linguistic background were identical to those of Experiment 1. Self-reported proficiency ratings confirmed that Tibetan proficiency was significantly higher than Chinese proficiency: overall level (Tibetan: *M* = 4.21, *SD* = 0.57; Chinese: *M* = 3.65, *SD* = 0.60), speaking (Tibetan: *M* = 4.34, *SD* = 0.62; Chinese: *M* = 3.66, *SD* = 0.67), listening (Tibetan: *M* = 4.37, *SD* = 0.55; Chinese: *M* = 3.84, *SD* = 0.70), reading (Tibetan: *M* = 4.24, *SD* = 0.64; Chinese: *M* = 3.81, *SD* = 0.76), and writing (Tibetan: *M* = 4.13, *SD* = 0.61; Chinese: *M* = 3.77, *SD* = 0.73). Participants also reported using Tibetan more frequently than Chinese in daily life (daily use percentage: Tibetan *M* = 68.36%; Chinese *M* = 31.64%). These data confirm that Tibetan was the dominant language for all participants. All participants were unaware of the specific experimental hypotheses (they were informed that the study concerned Tibetan reading comprehension). All provided written informed consent prior to the experiment and received monetary compensation upon completion. None had participated in Experiment 1.

#### 3.1.2. Design

A 2 (word frequency: high-frequency, low-frequency) × 2 (presentation type: identical, transposed) within-participant design was employed.

#### 3.1.3. Experimental Materials

(1) Selection and compilation of materials

The lexical materials for Experiment 2 were derived from the 72 two-character words used in Experiment 1. With reference to the Frequency Dictionary of Modern Tibetan (Lu & Gao, 2007), we initially determined that high-frequency words occurred more than 1000 times per 10 million, while low-frequency words occurred less than 500 times per 10 million, and we selected 72 matched pairs of high- and low-frequency words that were similar in meaning. Within each pair, the high- and low-frequency words were also matched on horizontal word length (high: 2–6 phonemes; low: 3–6 phonemes) and total word length (3–9 phonemes for both). Independent-samples *t*-test showed a significant difference in word frequency between high-frequency and low-frequency words, with high-frequency words having a significantly higher frequency, *t*(142) = 3.78, *p* < 0.001. No significant differences were found in either horizontal word length, *t*(142) = 0.28, *p* = 0.78, or total word length, *t*(142) = −0.37, *p* = 0.71. This validates the effectiveness of the frequency-based grouping, meeting the requirements of the experiment. Detailed properties of the lexical materials are provided in Table 6.

Subsequently, the morphemes of each word pair were transposed to form the stimuli for the transposed condition, ensuring that all transposed items were non-words. This yielded four sets of two-character nouns as target words: ① high-frequency identical; ② high-frequency transposed; ③ low-frequency identical; and ④ low-frequency transposed.

Finally, the four sets of target words were embedded into the 72 sentence frames from Experiment 1. For the transposed condition, we ensured that no morpheme could form an actual word with adjacent characters. This yielded 288 (4 × 72) Tibetan sentences, in which all parts except the target words were identical. All sentences were single-line declarative sentences, with horizontal sentence length of 21 to 39 phonemes and total sentence length of 29 to 62 phonemes. The sentences contained no semantic or syntactic ambiguities and no punctuation. An example of the materials is presented in Table 7.

(2) Evaluation and arrangement of materials

Fifteen Tibetan university students who did not participate in the formal experiment were invited to rate the difficulty and naturalness of the sentences on 5-point scales (difficulty: 1 = very easy, 5 = very difficult; naturalness:1 = very natural, 5 = very unnatural). The ratings indicated that the sentences were relatively easy and natural (difficulty: *M* = 2.13, *SD* = 1.10; naturalness: *M* = 1.92 *SD* = 0.90). Additionally, a separate group of 30 Tibetan university students completed a cloze test on the 72 sentence frames with the target words omitted. The average cloze predictability for high-frequency words was 3.2% (*SE* = 0.01), while that for low-frequency words was 2.6% (*SE* = 0.01). Thus, the final materials met the requirements of low difficulty, high naturalness, and low predictability. The formal experimental sentences had an average horizontal sentence length of 27.83 phonemes, and an average total sentence length of 41.18 phonemes.

As in Experiment 1, a Latin square design was used to distribute the four experimental conditions across four lists, preventing participants from reading the same sentence frame more than once. Each participant was assigned to read only one list, which consisted of 8 practice trials (2 per condition) followed by 72 experimental trials (18 per condition).

#### 3.1.4. Apparatus, Procedure, and Measures

The apparatus, procedure, and measures were the same as those used in Experiment 1.

### 3.2. Results

The mean comprehension accuracy across all participants was 84.25%, indicating that they could understand the sentences well. In reference to the previous literature (Cao et al., 2023; Li et al., 2022a), the criteria for the exclusion of data were the same as those in Experiment 1. In total, 7.81% of all trials were removed. Linear mixed models (LMM) were conducted using the lme4 package (Version 1.1-34; Bates et al., 2015) in R (Version 4.3.1; R Core Team, 2023) to analyze the data. Word frequency and presentation types were treated as fixed factors. Participants and items were treated as crossed random effects, with by-participant and by-item random intercepts and slopes. All other analytical steps were identical to those in Experiment 1. The final model structure for each eye-movement measure, along with the full model comparison results (including AIC values and likelihood ratio tests), is provided in Appendix A Table A2. Because closed-form power procedures for LMMs are not readily implemented in standard sample-size software (Kisilewicz et al., 2026), we further verified the adequacy of the sample size, especially for the interaction in Experiment 2. Referring to the previous literature (Chang et al., 2020; Chang et al., 2025; Pegado & Grainger, 2021), we used the simr package (Version 1.0.10; Green & MacLeod, 2016) in R to conduct a simulation-based power analysis based on the actual analysis model. The results showed that for a medium-sized effect (SESOI = 0.25), the sample size of Experiment 2 (*N* = 64) had sufficient statistical power (≥80%).

#### 3.2.1. Global Analysis

**Figure 4:**
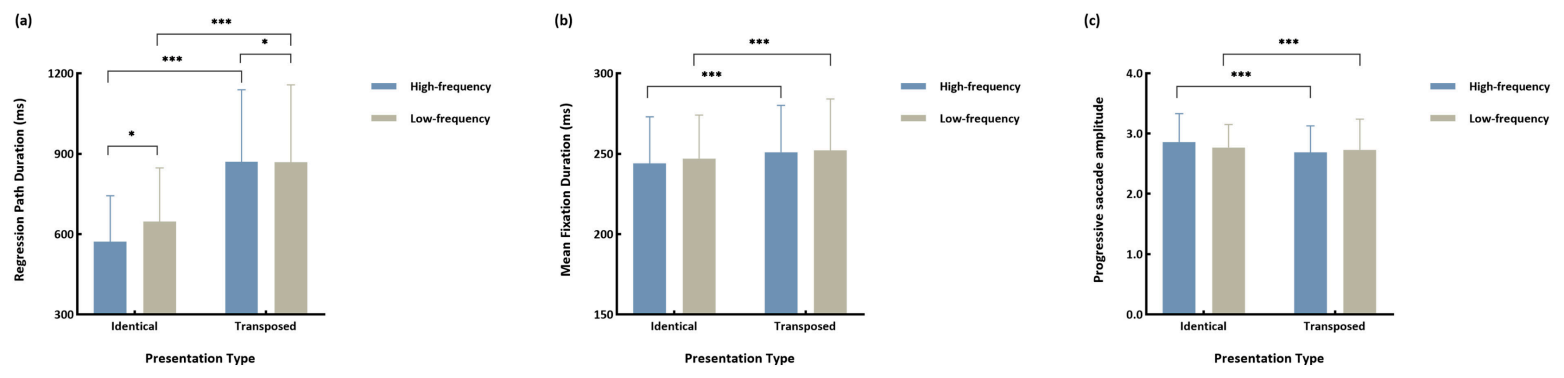
Statistical analysis results of eye-movement measures in global analysis of Experiment 2. (**a**) regression path duration, RPD; (**b**) mean fixation duration, MFD; (**c**) progressive saccade amplitude. *** *p* ≤ 0.001, * *p* ≤ 0.05.

Descriptive statistics of eye-movement measures under different conditions are shown in Table 8, with the corresponding statistical analysis results presented in Table 9 and Figure 4.

The global analyses revealed that, for RPD, the main effect of presentation type was significant, *t* = 9.99, *p* < 0.001, with the identical condition yielding shorter RPDs than the transposed condition. The main effect of word frequency was also significant, *t* = 2.28, *p* = 0.03, where high-frequency words elicited shorter RPDs than low-frequency words. The interaction between presentation type and word frequency was not significant, *t* = −1.68, *p* = 0.10.

For MFD, the main effect of presentation type was significant, *t* = 4.79, *p* < 0.001, with the identical condition yielding shorter MFDs than the transposed condition. The main effect of word frequency was not significant, *t* = 1.60, *p* = 0.12. The interaction between presentation type and word frequency was not significant, *t* = −1.01, *p* = 0.32.

For PSA, the main effect of presentation type was significant, *t* = −4.44, *p* < 0.001, with the identical condition yielding longer PSAs than the transposed condition. The main effect of word frequency was not significant, *t* = −1.04, *p* = 0.30. The interaction between presentation type and word frequency was not significant, *t* = 0.77, *p* = 0.45.

#### 3.2.2. Local Analysis

**Figure 5:**
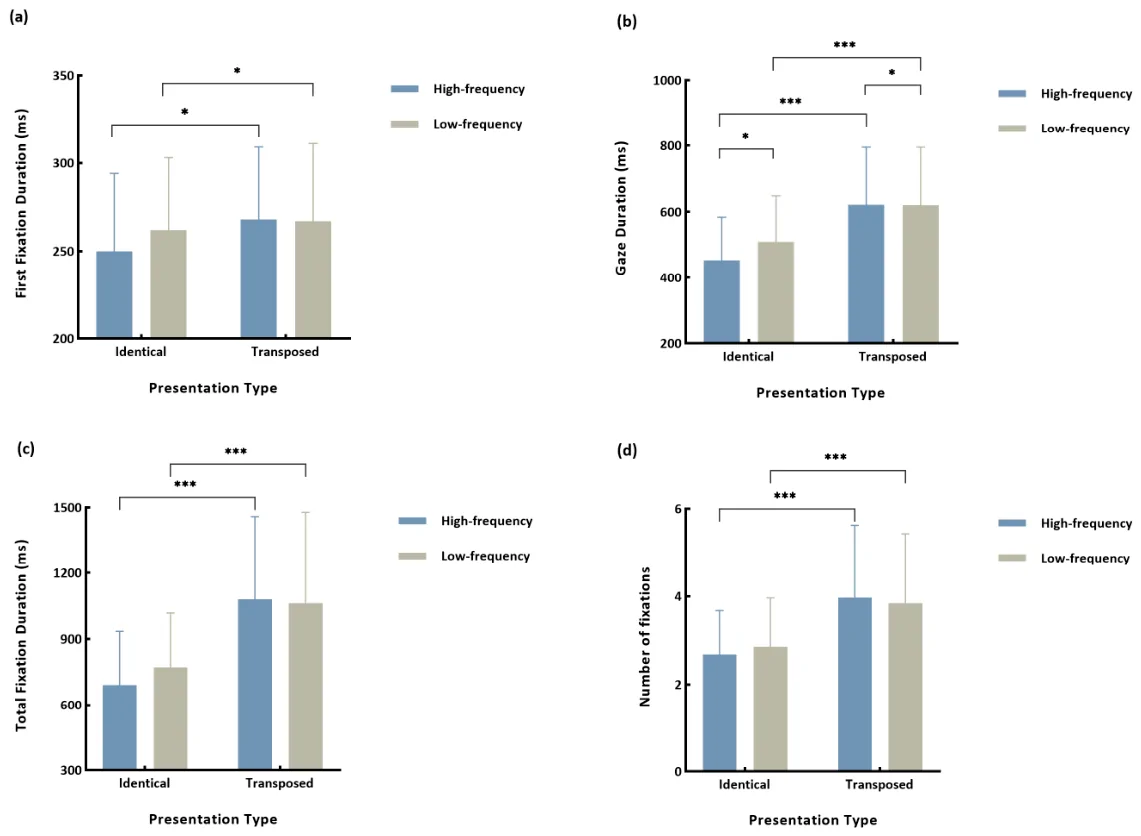
Statistical analysis results of eye-movement measures in local analysis of Experiment 2. (**a**) first fixation duration, FFD; (**b**) gaze duration, GD; (**c**) total fixation duration, TFD; (**d**) number of fixations, FC. *** *p* ≤ 0.001, * *p* ≤ 0.05.

Descriptive statistics of eye-movement measures under different conditions are shown in Table 10, with the corresponding statistical analysis results are presented in Table 11 and Figure 5.

The local analyses showed that, for FFD, the main effect of presentation type was significant, *t* = 2.13, *p* = 0.04, with the identical condition yielding shorter FFDs than the transposed condition. The main effect of word frequency was not significant, *t* = 1.71, *p* = 0.09. The interaction between presentation type and word frequency was not significant, *t* = −1.32, *p* = 0.19.

For GD, the main effect of presentation type was significant, *t* = 9.09, *p* < 0.001, with the identical condition yielding shorter GDs than the transposed condition. The main effect of word frequency was significant, *t* = 2.33, *p* = 0.02, where high-frequency words elicited shorter GDs than low-frequency words. The interaction between presentation type and word frequency was significant, *t* = −2.32, *p* = 0.02. The simple effect analysis revealed that, in the identical condition, high-frequency words elicited significantly shorter GDs than low-frequency words, *t* = 2.22, *p* = 0.03, while the frequency effect was not significant in the transposed condition, *t* = 0.13, *p* = 0.90.

For TFD, the main effect of presentation type was significant, *t* = 9.63, *p* < 0.001, with the identical condition yielding shorter TFDs than the transposed condition. The main effect of word frequency was not significant, *t* = 1.40, *p* = 0.17. The interaction between presentation type and word frequency was not significant, *t* = −1.98, *p* > 0.05.

For FC, the main effect of presentation type was significant, *t* = 8.84, *p* < 0.001, with the identical condition had fewer fixations than the transposed condition. The main effect of word frequency was not significant, *t* = 0.47, *p* = 0.64. The interaction between presentation type and word frequency was not significant, *t* = −1.83, *p* = 0.07.

#### 3.2.3. Bayesian Factor Analysis

In Experiment 2, seven eye-tracking measures were analyzed. Given the moderate to high correlations among these measures, we supplemented the key interaction effects (i.e., the non-significant interaction between word frequency and presentation type) with Bayesian factor analysis to provide additional robustness for statistical inference. Referring to the previous literature (Chang et al., 2020), this approach was applied to all temporal eye-movement measures to verify the reliability of the findings. The theoretical interpretation of this study is primarily based on consistent patterns across the main measures, rather than on a single significant result. The analysis was performed in R (Version 4.3.1; R Core Team, 2023), using the lmBF() function from the BayesFactor package (Version 0.9.12-4.4; Morey et al., 2025). Participants and items were included as random factors. The default Cauchy distribution served as the prior, with a scale factor of 0.5 for the g-prior, and the number of iterations was set to 100,000. Given the focus was on the interaction between the two independent variables in this experiment, the Bayes Factor *BF*_01_ (the subscript “_01_” representing H_0_ and H_1_) was computed by comparing two models: ① an additive model containing only the main effects of the word frequency and presentation type (corresponding to the null hypothesis, H_0_); and ② a full model that additionally included their interaction effect (the alternative hypothesis model, H_1_). *BF*_01_ quantifies how much more likely the observed data are under H_0_ than under H_1_. Generally, it is believed that when *BF*_01_ > 3, there is weak to moderate supporting evidence for H_0_; when *BF*_01_ > 10, there is strong supporting evidence for H_0_; and when *BF*_01_ < 1, it is considered that there is no difference between H_0_ and H_1_ (Vandekerckhove et al., 2014).

The Bayesian analyses provided varying levels of support for the additive model across measures. For FFD (*BF*_01_ = 26.23) and MFD (*BF*_01_ = 8.80), the evidence strongly favored the additive model over the interaction model. For RPD (*BF*_01_ = 3.09), the evidence was moderate. For GD (*BF*_01_ = 2.00) and TFD (*BF*_01_ = 1.63), the evidence was positive but weak, indicating that the data were consistent with the additive model but did not provide strong support. Notably, a significant interaction between word frequency and presentation type was observed for GD in the LMM. However, the *BF*_10_ values for GD and TFD (GD: *BF*_10_ = 0.50; TFD: *BF*_10_ = 0.61) indicated that the data did not support a reliable interaction, suggesting that the significant interaction for GD detected by the LMM does not appear to reflect a robust effect. Overall, the preponderance of evidence favored the additive model, although the strength of this support varied across measures. Moreover, none of the other eye-movement measures showed a significant interaction, and the overall pattern remained most consistent with the additive model.

In summary, the results of Experiment 2 revealed that, in the identical condition, all temporal eye-movement measures (RPD, MFD, FFD, GD, and TFD) were significantly shorter, PSA was larger, and FC was lower relative to the transposed condition. This pattern was consistent with the findings of Experiment 1. These results indicate that morpheme position information is actively processed during Tibetan reading, and incorrect morpheme position information disrupts lexical access, further supporting the important role of morpheme position coding in Tibetan word recognition. Secondly, the significant main effect of word frequency was observed for RPD and GD, with high-frequency words being processed faster than low-frequency words; and no such effect reached significance for the other measures. Moreover, the interaction between word frequency and presentation type was not significant on RPD, MFD, PSA, FFD, TFD, or FC, a pattern that was consistent with the predictions of the morpheme access view. The only exception was GD, where a significant interaction emerged. As discussed below, this interaction may reflect the role of morpheme position information in early lexical access, but its overall impact on the theoretical interpretation should be considered in light of the non-significant interactions in the other measures and the weak Bayesian evidence.

## 4. Discussion

The present study investigated how morpheme position information affects lexical processing during Tibetan reading through two eye-tracking experiments. The results demonstrated the following: Firstly, morpheme position information is actively processed in Tibetan reading, and incorrect morpheme position information significantly interferes with lexical processing. Secondly, there is the transposed-letter (TL) effect in Tibetan reading, indicating that nonwords with transposed letters activate the base word more readily than nonwords with substituted letters. Thirdly, the recognition of Tibetan two-character words aligns with the morpheme access view, suggesting that morpheme recognition plays a foundational role in lexical access. The following sections discuss these findings in detail.

### 4.1. The Processing of Morpheme Position Information in Tibetan Reading

Experiment 1 revealed a consistent ordinal pattern across all eye measures. Reading was fastest and most efficient in the identical condition (shortest RPD, MFD, FFD, GD, and TFD; largest PSA; and fewest fixations), intermediate in the transposed condition, and slowest and least efficient in the substituted condition (longest temporal measures; smallest PSA; and most fixations). This pattern indicates that morpheme position information is encoded during Tibetan sentence reading and that incorrect morpheme position information significantly disrupts lexical processing. In addition, performance in the transposed condition was significantly better than in the substituted condition (shorter RPD, MFD, FFD, GD, and TFD; larger PSA; and fewer fixations), indicating flexible processing of morpheme position information. The fact that transposed non-words activated the base word more readily than substituted non-words provides evidence for the existence of a TL effect in Tibetan reading. Experiment 2 showed a consistent trend with Experiment 1: the identical condition again yielded shorter RPD, MFD, FFD, GD, and TFD than the transposed condition, with larger PSA and fewer fixations. These results further demonstrate that morpheme position information is involved in the Tibetan word processing, and incorrect morpheme position information can interfere with Tibetan reading. Morpheme position information plays an important role in Tibetan word recognition.

The combined results of the two experiments indicate that Tibetan readers flexibly process morpheme position information and that a stable TL effect exists in Tibetan reading. This finding not only confirms the hypotheses of the present study but is also consistent with previous studies on the TL effect in both alphabetic writing systems and Chinese reading (Bian et al., 2010; Boudelaa et al., 2019; Cao et al., 2023; Gu et al., 2015; Lété & Fayol, 2013; Perea et al., 2010). For instance, Johnson et al. (2007) employed the boundary paradigm, manipulating parafoveal previews of target words as identical, transposed, or substituted letter strings (within-word or word-final letters; e.g., “clerk-celrk-cohrk-clekr-clefn”). They found that transposed non-words elicited a larger preview benefit than substituted non-words, indicating the existence of the TL effect during sentence reading. Similarly, Chang et al. (2020) reported flexible encoding of character positions in the parafoveal region during Chinese reading, further supporting the presence of the TL effect in Chinese reading.

The present findings demonstrate that Tibetan readers invested greater cognitive effort and reading time to comprehend sentences containing polymorphemic words with transposed morphemes, highlighting the critical role of morpheme position information in Tibetan lexical access. This suggests that morphemic information (including both identity and position information) is functional in Tibetan reading, with morpheme position information substantially affecting the reading process. Additionally, the TL effect observed in this study points to potential commonality in positional encoding across Tibetan, alphabetic, and Chinese writing systems, indicating a degree of cross-linguistic consistency in this phenomenon. Moreover, the significant effect of morpheme position information implies active processing at the morpheme level during Tibetan reading, which is inconsistent with a strict whole-word access view. Instead, the data align better with the morpheme access view or the dual-route access view, a premise that motivated the further investigation of lexical access representations in Tibetan two-character words in Experiment 2.

### 4.2. The Lexical Access and Representation of Polymorphemic Words in Tibetan Reading

In Experiment 2, the main effect of word frequency was significant only for RPD and GD. High-frequency words elicited significantly shorter RPD and GD than low-frequency words, whereas the frequency effect was not significant on other measures. GD reflects the early stage of lexical access and is sensitive to both pre-lexical and lexical features, serving as a key indicator of lexical processing efficiency. RPD encompasses the time spent on regressions and subsequent reprocessing due to initial integration difficulty, thus reflecting both early lexical integration and later, sentence-level re-analysis (Yan et al., 2013). The significant frequency effect on GD and RPD in this study suggests that word frequency primarily affects the early stage of lexical access and the initial sentence-level integration of words in Tibetan reading. The absence of a significant frequency effect on TFD and FC may indicate that readers adopted compensatory strategies (such as increased regressions and more fixations) to overcome the initial difficulty associated with low-frequency words, ultimately achieving sufficient comprehension of words with different frequencies. Furthermore, the unique orthographic features of Tibetan may also modulate the observed processing patterns. Tibetan, as an alphabetic script with logographic characteristics, features a dual structure where its text is composed of alphabetic phonemes, arranged in both linear and three-dimensional layouts. In Tibetan texts, word boundaries are not explicitly marked by spaces, but are instead signaled by the inter-character tsek “་” (Gao et al., 2020; Li et al., 2022a). Compared to letter transpositions in English or character transpositions in Chinese (e.g., “judge-jugde”, “复杂-杂复”), transposing Tibetan characters (e.g., “འཇའ་ཚོན་-ཚོན་འཇའ་”) induces more substantial spatial distortion of word forms and causes greater disruption to morphemic information, thereby imposing stronger interference on readers’ visual perception during word processing. In this context, compared to lower-level visual factors at the character level, word frequency—as a higher-level lexical factor (Kuperman et al., 2024; Li et al., 2022a)—may have its moderating effect partially attenuated under conditions of transposed morpheme. This could be an important reason why the word-frequency effect was not observed consistently across all eye-movement measures.

More importantly, the results of Experiment 2 revealed that the interaction between word frequency and presentation type was not significant for most measures. The one notable exception was GD, where a significant interaction was observed. Simple-effects analysis showed that the frequency effect was present in the identical condition but absent in the transposed condition. This pattern is consistent with the morpheme access view: when the morpheme order is preserved (identical condition), readers can efficiently access the morpheme representations, leading to a clear frequency effect; when the morpheme order is disrupted (transposed condition), the access to morpheme representations is hindered, and this frequency advantage disappears. Importantly, this interaction can be accommodated within the morpheme access view, as it suggests that even within this framework, morpheme position information can modulate the efficiency of lexical access at the earliest stage of processing. The fact that this interaction was limited to GD and was not supported by strong Bayesian evidence, suggests that it represents a relatively subtle modulation rather than a fundamental challenge to the additive model. Therefore, the overall pattern across measures remained most consistent with the morpheme access view. Taken together, the present findings indicate that readers process morpheme position information in Tibetan in a consistent manner regardless of word frequency, supporting the morpheme decomposition pathway for polymorphemic word recognition, i.e., the morpheme access view. During Tibetan reading, readers flexibly utilize morphemic information (both identity and position). Even when morpheme position is transposed, they can activate the correct morpheme identity information, subsequently re-encode and integrate the incorrect positional information, and ultimately access lexical meaning. This pattern implies that morpheme position information is actively processed during the recognition of Tibetan two-character words, and that morphemic processing contributes substantially to lexical access, which is most consistent with the morpheme access account. However, this does not rule out the existence of whole-word representations. Models involving contributions from both morphemic and whole-word levels remain possible and plausible.

In summary, from the perspective of morpheme position processing, the present study provides evidence that the lexical access representation of Tibetan polymorphemic words aligns with the morpheme access view, wherein both morpheme identity and position information jointly contribute to lexical recognition.

### 4.3. Limitations

This study presents the first direct investigation of how morpheme position information affects lexical processing during Tibetan reading. The results show that Tibetan readers actively utilize and flexibly encode morpheme position information, manifesting the robust TL effect. Additionally, the lexical access representation of Tibetan polymorphemic words aligns with the morpheme access view. Collectively, these findings provide new empirical evidence to resolve theoretical debates, enrich the relevant theoretical framework, and contribute to filling gaps in the field, holding both theoretical and practical relevance.

However, this study still has several limitations: First, the sample was limited to young, skilled Tibetan college students. Although all participants shared a similar Tibetan/Chinese bilingual background, the extent to which this specific bilingual experience influences lexical access remains unknown. Therefore, the findings may not generalize to developing readers, less-proficient readers, or populations with different language profiles. Second, the experimental materials were relatively simple, restricted to two-character nouns, and the sentences containing transposed or nonword items were experimentally constructed. This reduces the ecological validity and limits the generalizability of the results to natural reading contexts. Third, lexical access is influenced by multiple top-down and bottom-up factors beyond word frequency. Factors such as semantic transparency, which may modulate morpheme position processing, were not systematically examined in the present study. Fourth, the temporal relationship between the processing of morpheme identity and position information remains unexplored, as the present experimental paradigm does not offer sufficient evidence to reveal the time course of these processes. Finally, although the robust TL effect observed in this study is consistent with the morpheme access view, the current design cannot conclusively discriminate among the three proposed lexical access models; multimodal evidence with higher temporal and neural resolution (e.g., ERP, fNIRS, fMRI) would be required to fully resolve this issue.

## 5. Conclusions

This study examined the role of morpheme position information in Tibetan lexical processing through two experiments, and the main conclusions are as follows: (1) Morpheme position information is processed during Tibetan reading, and the transposed morpheme significantly affect reading, demonstrating a stable transposed-letter (TL) effect. (2) During the recognition of Tibetan polymorphemic words, the processing of morphemic information plays an important role in lexical access, supporting the morpheme access view.

## Figures and Tables

**Table 1:** Example of sentence material for Experiment 1.

Presentation Type	Sentence (Corresponding English Translation)
Identical	བོད་ཡིག་རྒན་ལགས་ཀྱི་**གསུང་རྩོམ་**ལ་བྱ་དགའ་ཨང་དང་པོ་ཐོབ། (The Tibetan language teacher’s **work** won first place.)
Transposed	བོད་ཡིག་རྒན་ལགས་ཀྱི་**རྩོམ་གསུང་**ལ་བྱ་དགའ་ཨང་དང་པོ་ཐོབ། (The Tibetan language teacher’s **wrok** won first place.)
Substituted	བོད་ཡིག་རྒན་ལགས་ཀྱི་**དབུལ་སྨོན་**ལ་བྱ་དགའ་ཨང་དང་པོ་ཐོབ། (The Tibetan language teacher’s **wuvk** won first place.)

Note: The target words are bolded but are presented normally during the experiment. The English examples are provided solely to illustrate the three presentation types and do not correspond perfectly in meaning to the original Tibetan contexts.

**Table 2:** The mean and standard deviations of eye-movement measures in the global analysis of Experiment 1.

Presentation Type	RFD	MFD	PSA
Identical	611 (191)	245 (27)	2.80 (0.31)
Transposed	894 (334)	250 (28)	2.72 (0.33)
Substituted	984 (347)	254 (28)	2.63 (0.30)

Note: The standard deviations are in parentheses. RFD = regression path duration (ms); MFD = mean fixation duration (ms); PSA = progressive saccade amplitude (number of horizontal phonemes).

**Table 3:** Statistical analysis results of eye-movement measures in the global analysis of Experiment 1.

Measures	Fixed Effect	*b*	*SE*	*t*	95%CI	
					2.5%	97.5%
RPD	Intercept	6.50	0.04	148.16 ***	6.41	6.58
	Transposed–Identical	0.36	0.04	9.55 ***	0.28	0.43
	Substituted–Transposed	0.12	0.04	3.16 **	0.04	0.19
	Substituted–Identical	0.48	0.04	12.73 ***	0.40	0.55
MFD	Intercept	5.51	0.01	394.15 ***	5.48	5.54
	Transposed–Identical	0.02	0.01	2.97 **	0.01	0.03
	Substituted–Transposed	0.02	0.01	2.35 *	0.003	0.03
	Substituted–Identical	0.04	0.01	5.32 ***	0.02	0.05
PSA	Intercept	0.42	0.02	18.15 ***	0.37	0.46
	Transposed–Identical	−0.03	0.02	−2.02 *	−0.07	−0.001
	Substituted–Transposed	−0.04	0.02	−2.13 *	−0.07	−0.003
	Substituted–Identical	−0.07	0.02	−4.13 ***	−0.10	−0.04

Note: RPD = regression path duration; MFD = mean fixation duration; PSA = progressive saccade amplitude. *** *p* ≤ 0.001, ** *p* ≤ 0.01, * *p* ≤ 0.05; CI = confidence interval. The same applies below.

**Table 4:** The mean and standard deviations of eye-movement measures in the local analysis of Experiment 1.

Presentation Type	FFD	GD	TFD	FC
Identical	260 (45)	490 (119)	658 (211)	2.50 (0.86)
Transposed	272 (41)	640 (180)	1053 (352)	3.79 (1.43)
Substituted	275 (40)	714 (199)	1247 (468)	4.47 (1.82)

Note: FFD = first fixation duration (ms); GD = gaze duration (ms); TFD = total fixation duration (ms); FC = number of fixations. The same applies below.

**Table 5:** Statistical analysis results of eye-movement measures in the local analysis of Experiment 1.

Measures	Fixed Effect	*b*	*SE*	*t*	95%CI	
					2.5%	97.5%
FFD	Intercept	5.52	0.02	302.34 ***	5.48	5.55
	Transposed–Identical	0.04	0.02	2.03 *	0.001	0.08
	Substituted–Transposed	0.01	0.02	0.65	−0.03	0.05
	Substituted–Identical	0.05	0.02	2.69 **	0.01	0.09
GD	Intercept	6.23	0.03	193.38 ***	6.17	6.30
	Transposed–Identical	0.24	0.03	7.67 ***	0.18	0.30
	Substituted–Transposed	0.11	0.03	3.51 ***	0.05	0.17
	Substituted–Identical	0.35	0.03	11.20 ***	0.29	0.42
TFD	Intercept	6.68	0.04	149.17 ***	6.59	6.76
	Transposed–Identical	0.47	0.04	12.23 ***	0.40	0.55
	Substituted–Transposed	0.17	0.04	4.40 ***	0.09	0.25
	Substituted–Identical	0.64	0.04	16.65 ***	0.57	0.72
FC	Intercept	1.13	0.04	28.15 ***	1.05	1.21
	Transposed–Identical	0.40	0.03	11.96 ***	0.34	0.47
	Substituted–Transposed	0.15	0.03	4.40 ***	0.08	0.21
	Substituted–Identical	0.55	0.03	16.39 ***	0.49	0.62

Note: *** *p* ≤ 0.001, ** *p* ≤ 0.01, * *p* ≤ 0.05.

**Table 6:** Properties of high- and low-frequency word pairs in Experiment 2.

Word Frequency	Horizontal Word Length	Total Word Length	Log_10_ (Word Frequency)
High	4.25 (0.92)	6.21 (1.39)	2.66 (0.64)
Low	4.21 (0.87)	6.29 (1.26)	1.81 (0.81)

Note: The data are presented as *M* (*SD*).

**Table 7:** Example of sentence material for Experiment 2.

Presentation Type	Sentence (Corresponding English Translation)
High-frequency Identical	བོད་ཡིག་རྒན་ལགས་ཀྱི་**རྩོམ་རིག་**ལ་བྱ་དགའ་ཨང་དང་པོ་ཐོབ། (The Tibetan language teacher’s **text** won first place.)
High-frequency Transposed	བོད་ཡིག་རྒན་ལགས་ཀྱི་**རིག་རྩོམ་**ལ་བྱ་དགའ་ཨང་དང་པོ་ཐོབ། (The Tibetan language teacher’s **txet** won first place.)
Low-frequency Identical	བོད་ཡིག་རྒན་ལགས་ཀྱི་**གསུང་རྩོམ་**ལ་བྱ་དགའ་ཨང་དང་པོ་ཐོབ། (The Tibetan language teacher’s **work** won first place.)
Low-frequency Transposed	བོད་ཡིག་རྒན་ལགས་ཀྱི་**རྩོམ་གསུང་**ལ་བྱ་དགའ་ཨང་དང་པོ་ཐོབ། (The Tibetan language teacher’s **wrok** won first place.)

Note: The target words are bolded but are presented normally during the experiment.

**Table 8:** The mean and standard deviations of eye-movement measures in the global analysis of Experiment 2.

Word Frequency	Presentation Type	RPD	MFD	PSA
High	Identical	572 (171)	244 (29)	2.86 (0.47)
	Transposed	870 (269)	251 (29)	2.69 (0.44)
Low	Identical	647 (200)	247 (27)	2.77 (0.38)
	Transposed	868 (289)	252 (32)	2.73 (0.51)

**Table 9:** Statistical analysis results of eye-movement measures in the global analysis of Experiment 2.

Measures	Fixed Effect	*b*	*SE*	*t*	95%CI	
					2.5%	97.5%
RFD	Intercept	6.38	0.04	159.01 ***	6.30	6.46
	Word Frequency	0.07	0.03	2.28 *	0.01	0.13
	Presentation Type	0.35	0.04	9.99 ***	0.28	0.42
	Word Frequency × Presentation Type	−0.10	0.06	−1.68	−0.22	0.02
MFD	Intercept	5.50	0.01	380.08 ***	5.47	5.53
	Word Frequency	0.01	0.004	1.60	−0.002	0.02
	Presentation Type	0.02	0.004	4.79 ***	0.01	0.03
	Word Frequency × Presentation Type	−0.01	0.01	−1.01	−0.03	0.01
PSA	Intercept	0.43	0.03	15.47 ***	0.37	0.48
	Word Frequency	−0.01	0.01	−1.04	−0.04	0.01
	Presentation Type	−0.06	0.01	−4.44 ***	−0.08	−0.03
	Word Frequency × Presentation Type	0.02	0.02	0.77	−0.03	0.07

Note: *** *p* ≤ 0.001, * *p* ≤ 0.05.

**Table 10:** The mean and standard deviations of eye-movement measures in the local analysis of Experiment 2.

Word Frequency	Presentation Type	FFD	GD	TFD	FC
High	Identical	250 (44)	453 (131)	691 (243)	2.68 (0.99)
	Transposed	268 (41)	622 (173)	1079 (379)	3.97 (1.65)
Low	Identical	262 (41)	510 (138)	772 (246)	2.86 (1.10)
	Transposed	267 (44)	620 (175)	1062 (416)	3.84 (1.59)

**Table 11:** Statistical analysis results of eye-movement measures in the local analysis of Experiment 2.

	Fixed Effect	*b*	*SE*	*t*	95%CI	
					2.5%	97.5%
FFD	Intercept	5.49	0.02	321.27 ***	5.46	5.53
	Word Frequency	0.03	0.01	1.71	−0.004	0.05
	Presentation Type	0.03	0.01	2.13 *	0.002	0.06
	Word Frequency × Presentation Type	−0.04	0.03	−1.32	−0.09	0.02
GD	Intercept	6.13	0.03	186.61 ***	6.07	6.20
	Word Frequency	0.06	0.03	2.33 *	0.01	0.11
	Presentation Type	0.23	0.03	9.10 ***	0.18	0.28
	Word Frequency × Presentation Type	−0.11	0.05	−2.32 *	−0.20	−0.02
TFD	Intercept	6.58	0.04	148.21 ***	6.49	6.67
	Word Frequency	0.05	0.04	1.40	−0.02	0.12
	Presentation Type	0.39	0.04	9.63 ***	0.31	0.47
	Word Frequency × Presentation Type	−0.14	0.07	−1.98 ^•^	−0.27	−0.001
FC	Intercept	1.06	0.04	26.20 ***	0.98	1.14
	Word Frequency	0.01	0.03	0.47	−0.05	0.08
	Presentation Type	0.32	0.04	8.84 ***	0.25	0.39
	Word Frequency × Presentation Type	−0.11	0.06	−1.83	−0.23	0.01

Note: *** *p* ≤ 0.001, * *p* ≤ 0.05, ^•^ *p* ≤ 0.1.

## Data Availability

The original contributions presented in the study are available from the corresponding authors upon reasonable request.

## Appendix A

**Table A1:** Model comparison results for eye-movement measures in Experiment 1.

Measures	AIC			*p*		
	Model 1	Model 2	Model 3	Model 1 vs. Model 2	Model 2 vs. Model 3	Model 3 vs. Model 1
RPD	6862	6854	6864	0.85	0.001	0.02
MFD	−6673	−6680	−6679	0.77	0.05	0.2
PSA	6659	6648	6652	1.00	0.02	0.19
FFD	3621	3616	3607	0.45	0.87	0.77
GD	6967	6960	6955	0.73	0.37	0.61
TFD	6421	6415	6442	0.61	<0.001	<0.001
FC	6061	6055	6101	0.65	<0.001	<0.001

Note: Model 1 = ~ presentation type + (1 + presentation type | participant) + (1 + presentation type| item), the maximal model, including random intercepts for participants and items, as well as random slopes for presentation type; Model 2 = ~ presentation type + (1 + presentation type | participant) + (1 | item), retaining the participant-level random slope and removing the item-level random slope; Model 3 = ~ presentation type + (1 | participant) + (1 | item), retaining only random intercepts. All measures were analyzed using Model 3, as Model 1 and Model 2 yielded singular fits. Model comparison was conducted using a series of likelihood ratio tests (LRT). The *p*-values indicate the significance of the model comparison against the specified model. AIC = Akaike’s information criterion. The same applies below.

**Table A2:** Model comparison results for eye-movement measures in Experiment 2.

Measures	AIC		*p*	Final Model Description
	Maximal Model	Final Model		
RPD	7601	7578	0.90	~ fq * pt + (1 + pt | participant) + (1 | item)
MFD	−6310	−6290	0.54	~ fq * pt + (1 | participant) + (1 | item)
PSA	6138	6118	0.94	~ fq * pt + (1 + fq + pt | participant) + (1 | item)
FFD	3694	3665	1.00	~ fq * pt + (1 + fq | participant) + (1 | item)
GD	7309	7293	0.73	~ fq * pt + (1 + fq + pt | participant) + (1 | item)
TFD	7304	7285	0.87	~ fq * pt + (0 + fq + pt | participant) + (1 | item)
FC	6833	6892	0.77	~ fq * pt + (0 + fq + pt | participant) + (1 | item)

Note: fq = word frequency; pt = presentation type. Maximal Model = ~ fq * pt + (1 + fq * pt | participant) + (1 + fq * pt | item). The random-effects structure was progressively simplified from the Maximal Model until a convergent, non-singular model was achieved (the Final Model). All measures were analyzed using the Final Model. The *p*-values indicate the significance of the comparison of the Maximal Model against the Final Model.
